# Epidemiology, Virology, and Mutation Landscape of Monkeypox Virus From Past to Present

**DOI:** 10.7759/cureus.67872

**Published:** 2024-08-26

**Authors:** Suganandhini Mani, Alagammai Ganesan, Thirumalai Arunagiri, Vamsi Ravi Kumaran, Kanaka Parvathi Kannaiah, Chitra Vellapandian, Hemanth Kumar Chanduluru

**Affiliations:** 1 Pharmacy, SRM College of Pharmacy, SRM Institute of Science and Technology, Kattankulathur, IND

**Keywords:** monkeypox virus, monkeypox, mpxv, poxviridae infection, epidemiology of mpox, orthopoxvirus, mpox

## Abstract

Monkeypox (Mpox) has emerged as a significant threat to the global population. Initially identified in a rural area of Africa in 1970, after the eradication of smallpox, it spread rapidly across various African nations. The ongoing evolution of the monkeypox virus (MPXV), which causes Mpox, and its potential for cross-species transmission led to a global outbreak in 2022. Despite the control measures during the outbreak, Mpox cases continue in several African nations, posing a persistent public health threat. Global surveillance is crucial to eradicating MPXV from human populations and preventing its resurgence. Factors contributing to MPXV's increased transmissibility and immune evasion include its mutation rate, adaptability, and genetic evolution. Therefore, understanding the epidemiology and virology of Mpox is essential for developing effective prevention and control strategies. This study explores the history of human Mpox, the complexity of MPXV, how it replicates, and drug-resistant mutations. It will also stress how important it is to study how the circadian clock affects virus replication in infectious diseases in order to effectively fight this new public health threat. Understanding these aspects is crucial for developing effective strategies against Mpox as well as addressing the challenges posed by genetic mutations and resistance. The compiled information in this review underscores the critical need for continued research and monitoring to tackle the evolving dynamics of Mpox and its broader impact on global health.

## Introduction and background

During the development of the polio vaccine in 1958, monkeypox (Mpox) was initially identified in monkeys, which are used for vaccine research. Since then, it has been spotted in several animal reservoirs, most notably in rodents and minor mammal species [1]. The Democratic Republic of the Congo (DRC) (formerly Zaire) was the site of the first human Mpox infection discovery in 1970. Consequently, Africa's Central and Western regions became the hotspots for Mpox due to the high density of tropical rainforests and organisms that could spread the virus [2]. The United States saw the first known case of Mpox outside of Africa about 30 years later as a result of zoonotic transmission of the virus from an infected animal. In the later decades, outbreaks of Mpox have occurred in several non-African regions [1]. But in 2022, the Mpox outbreak spread internationally, and as a result, even though there were no travel restrictions, it was deemed a global health emergency. The World Health Organization (WHO) proposed "Mpox" as a term to replace "monkeypox" on November 28, 2022 [3].

The viral and zoonotic disease Mpox, which evolved following the elimination of smallpox [4], is caused by the monkeypox virus (MPXV) [5], and it has two distinct genetic clades, one from the Congo Basin and the other originating from West Africa [6]. These genetic clades were later renamed as Clade I and Clade II, respectively [7]. The viral nature of Mpox makes it susceptible to transmission from animals to humans. Additionally, individuals can also spread it to one another [8]. Apart from lymphadenopathy, symptoms of Mpox are comparable to those of smallpox, which range from sickness to cough, muscle pain, and rashes during the incubation period of four to 21 days [9]. Antivirals such as tecovirimat, brincidofovir, and cidofovir, as well as vaccinia immune globulin (VIG), are available medications, even though the majority of Mpox cases occur as a reversible disease for which supportive care usually suffices. Patients with severe illnesses or compromised immune systems can benefit from antivirals [10].

Smallpox vaccines, antivirals, and VIG can control an outbreak of Mpox, although there is currently no effective gunshot treatment for the virus. The percentage of people who were protected against MPXV by prior smallpox vaccinations was 85% [11]. Smallpox vaccinations, such as ACAM2000 and JYNNEOS, are efficient against MPXV [12]. This study provides a comprehensive review of the historical and contemporary epidemiological data on human Mpox in both endemic and non-endemic regions. It also delves into the complexities of MPXV, its replication mechanisms, the impact of biological cycles on viral replication, and the implications of drug-resistant mutations. These findings could provide valuable insights into the ongoing control and prevention of Mpox, as well as guide future research and public health efforts.

Epidemiology of Mpox

Two breakouts of a non-fatal pox-like illness in cynomolgus monkeys were seen in the monkey colony at the Statens Serum Institute located in Copenhagen, Denmark, in 1958, which led to the discovery of MPXV. To research and produce polio vaccines, the Institute typically receives a steady supply of monkeys via air from Singapore [13]. Since the virus originated from an outbreak among captive monkeys, it is named “monkeypox” [14].

**Figure 1:**
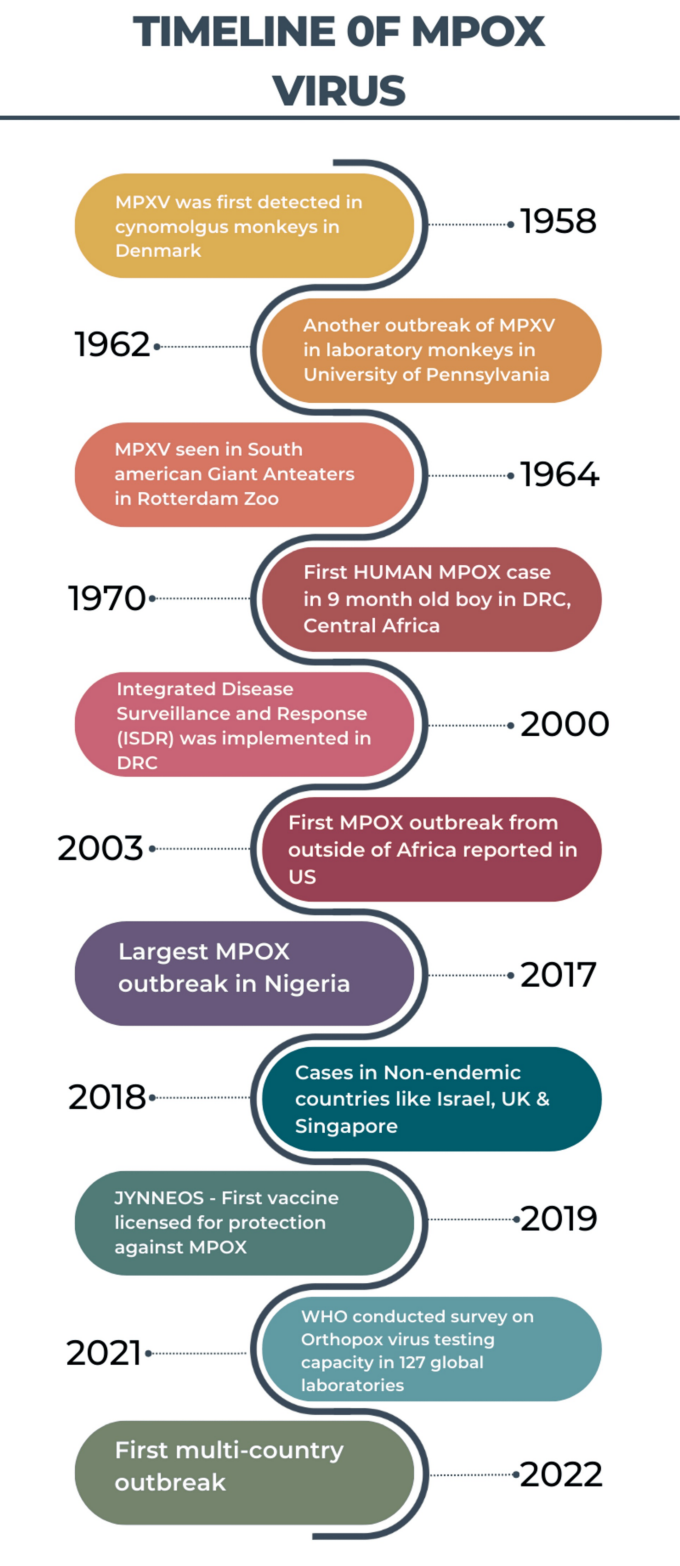
Timeline of MPXV: The above timeline effectively elucidates the history of MPXV over the years, from its initial detection in 1958 until the global outbreak in 2022. MPXV: monkeypox virus, DRC: Democratic Republic of the Congo, Mpox: monkeypox, UK: United Kingdom, WHO: World Health Organization Created using BioRender

Following the eradication of smallpox in 1970, it was discovered that infections resembling smallpox persistently occurred in rural areas of Africa. This observation led to the designation of Mpox as a distinct disease [15]. The DRC includes 26 provinces, of which 11 are known to be endemic for Mpox. However, in recent years, both the overall number of Mpox cases and the provinces reporting Mpox have increased, reaching 22 provinces as of November 2023. Although several mammals, including monkeys and squirrels, are known to be susceptible, the virus's natural reservoir is unknown, and these animals have rarely been connected to outbreaks [16]. The history of MPXV was elucidated through a timeline chart in Figure 1.

## Review

History of human Mpox

Endemic Countries

Both regions of West Africa and Central Africa are endemic for Mpox; however, prior to the 2022 outbreak, a small number of cases were discovered away from Africa and were linked to imports from endemic nations [17]. In the DRC, Central Africa, a baby who had smallpox-like outbreaks was found to have very initial human Mpox in 1970. The kid experienced fever, headaches, and pus-filled blister eruptions, all of which were indicative of smallpox. In six African nations, the DRC, Cameroon, Cote d'Ivoire, Liberia, Nigeria, and Sierra Leone, a total of 48 confirmed and suspected cases of Mpox were reported during the 1970s. The majority of cases were reported in the DRC [18]. With a rise in cases from 38 in 1970-1979 to 511 in 1990-1999, the DRC was the most severely impacted nation. The loss of immunity following smallpox vaccination and greater human intrusion into sylvatic environments are blamed for the increase in incidence in Africa [19].

The count of confirmed and suspected cases of Mpox in the DRC increased ninefold between the 1970s and 1980s. Four additional African nations shared 14 additional cases. The DRC recorded 511 verified probable and/or suspected cases of Mpox, and Gabon reported nine confirmed cases. Cases continued to rise throughout the 1990s. Three African nations recorded cases of Mpox between 2000 and 2009; however, seven African countries (Cameroon, Central African Republic, DRC, Liberia, Nigeria, Sierra Leone, and Republic of the Congo) reported instances between 2010 and 2019 [18].

Despite elevated case fatality rates (CFR), the Congo Basin is still endemic in the DRC. Every year, 1000 cases are reported in the DRC [20]. Mpox instances have been documented since 2003 in several nations, with Nigeria seeing the biggest outbreak in 2017 [15]. Prior to 2017, the most recent instance of human Mpox in Nigeria had been documented in 1978. A significant Clade II outbreak by mid-November 2017 produced 42 laboratory-confirmed cases and 146 suspected cases from 14 states [21]. Thirteen endemic Western and Central African nations reported 1205 cases of Mpox between January 2022 and January 2023 [22].

Non-endemic Countries

US outbreak in 2003: In 2003, pet prairie dogs from Ghana infected 47 people in America, resulting in the first Mpox outbreak outside of Africa [22]. Given that most of the affected individuals fell ill after coming into contact with pet prairie dogs, it was deemed that native prairie dogs kept in cages with imported rats from Ghana in West Africa were the main origin of the outbreak [15].

The outbreak, which affected Illinois, Indiana, Kansas, Missouri, and Wisconsin states, resulted in 47 patients, of whom 37 had confirmed cases of MPXV infection and 10 had probable cases. With a few notable exceptions, the outbreak's illness manifestations were generally mild. Age and immunization status did not appear to have much of an impact on the clinical illness manifestation, and there were no deaths or instances of person-to-person transmission [23].

The majority of US outbreak patients, in contrast to those in Africa, had minor, self-limited febrile eruptions and illnesses. The strain of the virus that caused the mild disease manifestation and lack of fatalities was identified as Clade II through genetic analysis. A significant proportion of cases in the US outbreak were in adults, in contrast to Mpox incidents in Africa that significantly impacted children [23]. Following the 2003 outbreak in the US, Mpox has gained international attention as a serious public health concern [15].

Global outbreak in 2022: An international Clade IIb MPXV epidemic began in 2022 and continued to spread to many non-African nations that had never before reported an outbreak of Mpox [16]. As of January 1, 2022, 42 member states from five WHO regions, the Americas, Africa, Europe, Eastern Mediterranean, and Western Pacific have reported Mpox cases to the WHO, indicating a global outbreak [24]. An individual from an endemic region of Africa initially contracted the infection, which then spread to North America and Europe [20]. One hundred three member states have reported to the WHO that between January 1, 2022, and September 15, 2022, they recorded over 60,320 laboratory-confirmed cases of Mpox and 23 deaths [25].

Reports of MPXV infection were received on May 18, 2022, from Spain, Portugal, and Canada, in the order of seven, 14, and 13, respectively. The first cases of Mpox were confirmed in Italy, Sweden, and Belgium on May 19, 2022. Australia announced two cases on May 20, 2022. Recent returns from Europe were made by both patients. The first instances of Mpox were confirmed on May 20, 2022, by Germany, France, and the Netherlands, and on May 21, 2022, by Switzerland and Israel. In 103 traditionally non-endemic countries, over 84,000 cases of Mpox have been reported between May 2022 and January 2023 [22]. A mandatory 21-day quarantine for Mpox was first implemented in Belgium [20].

Even though epidemiological investigations are still in progress, most of the cases in the latest epidemic were mentioned through sexual wellness or other medical care in primary or secondary healthcare settings, with a previous record of travel that has primarily been to countries in Europe, North America, or other regions rather than those where the virus hasn't been previously identified [24]. Numerous Mpox incidents and clusters have never before been reported simultaneously in numerous nations across various WHO regions. Although the current outbreak has not resulted in a high death toll, the WHO considers the risk to be moderate worldwide [26]. The WHO director-general declared that the multicountry Mpox outbreak was a public health emergency of international concern (PHEIC) on July 23, 2022, following the second conference of the International Health Regulations (2005) Emergency Committee [25]. Regional outbreaks of Mpox, along with their outbreak period, count of reported cases, and a notable fact regarding the outbreak, are described in Table 1.

**Table 1:** Mpox outbreaks by region Mpox: monkeypox, DRC: Democratic Republic of the Congo, WHO: World Health Organization, PHEIC: public health emergency of international concern

Region	Outbreak period	Reported cases	Reported deaths	Notable information
West and Central Africa	1970s to present	Varies	Varies	Endemic region with periodic outbreaks which has DRC as the most affected country in Central Africa
United States	2003	47	0	Non-endemic region which experienced very initial Mpox outbreak outside of Africa. It is linked to pet prairie dogs from Ghana, Africa
Global	2022-2023	92,167 (as of December 1, 2023)	170	Rapid spread in non-endemic countries made WHO declare the outbreak as PHEIC

Recent epidemiological updates of Mpox outbreaks

The DRC is seeing the highest known spike in Mpox cases. Over 20,000 suspected cases of Mpox and over 1,000 fatalities have been recorded by the DRC since January 1, 2023. Additionally, in 2023, Mpox was reported in previously unreported geographic areas like South Kivu, Kwango Province, and Kinshasa. It is still unknown why this expansion, which affects men, women, and children, is occurring [16].

Recently, there has been an upsurge in probable cases of Clade I MPXV, which causes more severe sickness than Clade II MPXV, between 2023 and 2024. If this upsurge is not quickly contained, then there is a potential danger of worldwide dissemination. This panic is supported by reports of an increasing incidence of Mpox in certain neighboring countries where MPXV is endemic, notably the Republic of the Congo, where 19 cases have been verified. DRC reported numerous provincial-level outbreaks from January 1, 2023, to April 14, 2024, culminating in 19,919 probable cases of Clade I Mpox and 975 fatalities. Of the 26 provinces in the DRC, 25 of them reported cases of Clade I Mpox in 2023 and 2024, while the capital city of Kinshasa reported instances for the first time [27].

Cases of Mpox have been reported to WHO by 116 Member States in all six WHO regions since January 1, 2022. As of June 30, 2024, WHO had received reports of 99,176 laboratory-confirmed illnesses, 535 probable cases, and 208 deaths [28]. The WHO Director-General declared the current outbreak to be PHEIC on August 14, 2024, due to the recent spike in Mpox cases. This PHEIC determination for Mpox is the second in the last two years [29]. The recent rise in instances of Mpox poses a global threat to the dissemination of the disease. Hence, enhanced preventive measures and global surveillance are necessary to eradicate MPXV from human populations and stop it from resurfacing.

Virology of MPXV

**Figure 2:**
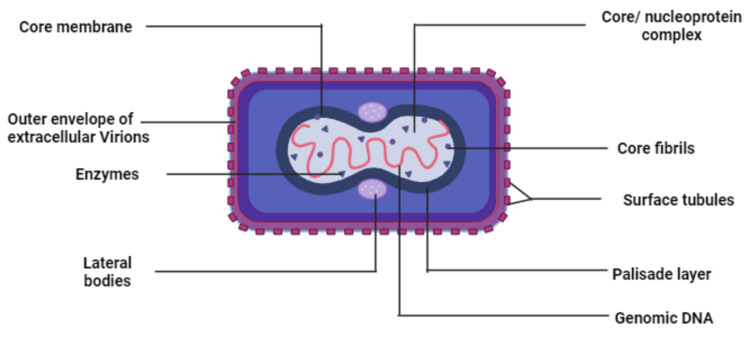
Illustration of MPXV: The structure of MPXV appears as oval or barrel-shaped particles. The virion primarily contains genomic DNA, enzymes, and core fibrils, which are tightly packed inside a core membrane. The core membrane has lateral bodies on two sides. There are two separate infectious forms of MPXV: IMV and EEV, which are differentiated by variations in surface glycoprotein. Finally, an outer envelope composed of a single lipid bilayer shields the tightly packed core in the IMV, while the EEV has an additional external membrane in which surface tubules are embedded. MPXV: monkeypox virus, DNA: deoxyribonucleic acid, IMV: intracellular mature virus, EEV: extracellular enveloped virus Created using BioRender

The double-stranded DNA virus MPXV is a member of the Poxviridae family, specifically the genus *Orthopoxvirus* (OPV), and based on their morphology, MPXV resembles other OPVs. They are ovoid or brick-shaped particles that are encased in an exterior membrane of lipoprotein that is geometrically corrugated. The outer membrane shields a tightly packed core that contains transcription factors, a double-stranded DNA genome, and enzymes, along with the core fibrils [2]. The structure of MPXV is clearly illustrated in Figure 2 [3].

The size of poxviruses, including Mpox, makes it difficult for them to quickly replicate and effectively evade host defenses. Due to their greater susceptibility to the host's immune system, poxviruses are more likely to trigger a defense response from the host. Poxviruses have developed various kinds of virulence factors to evade detection [5]. Linear double-stranded DNA with inverted tandem repeats (ITR) and a covalently closed hairpin on both the 5′ and 3′ ends make up the massive genome of MPXV [3].

With over 190 open reading frames (ORFs), the MPXV genome is roughly 197,000 kb in size. While the genome contains a large number of ORFs, around 30-50 are typically most critical for the infection process and pathogenicity of the virus [30]. Genes known to be necessary for OPVs are found in the central region of the MPXV genome [30]. The diverse ends of the genome, which contain ITR, flank the heavily conserved central coding region. Poxvirus replication and morphogenesis require a minimum of 90 ORFs. Various so-called non-essential ORFs, many of which have not yet been functionally characterized, contribute to variations in poxvirus host tropism, immunomodulation, and pathogenesis [31].

The characteristic dimensions of MPXV are approximately 280 nm × 220 nm, which gives rise to a barrel or oval shape. These virions comprise 30 structural and membrane viral proteins, forming a unique composition [32]. MPXV presents itself in two distinct infectious forms: the intracellular mature virus (IMV) and the extracellular enveloped virus (EEV). These two forms exhibit discernible variations in surface glycoproteins and methods of cell infection, highlighting the virus's remarkable adaptability [33]. The intricate virological features of MPXV continue to be a focus of ongoing research and investigation in the field of virology.

Clades of MPXV

**Figure 3:**
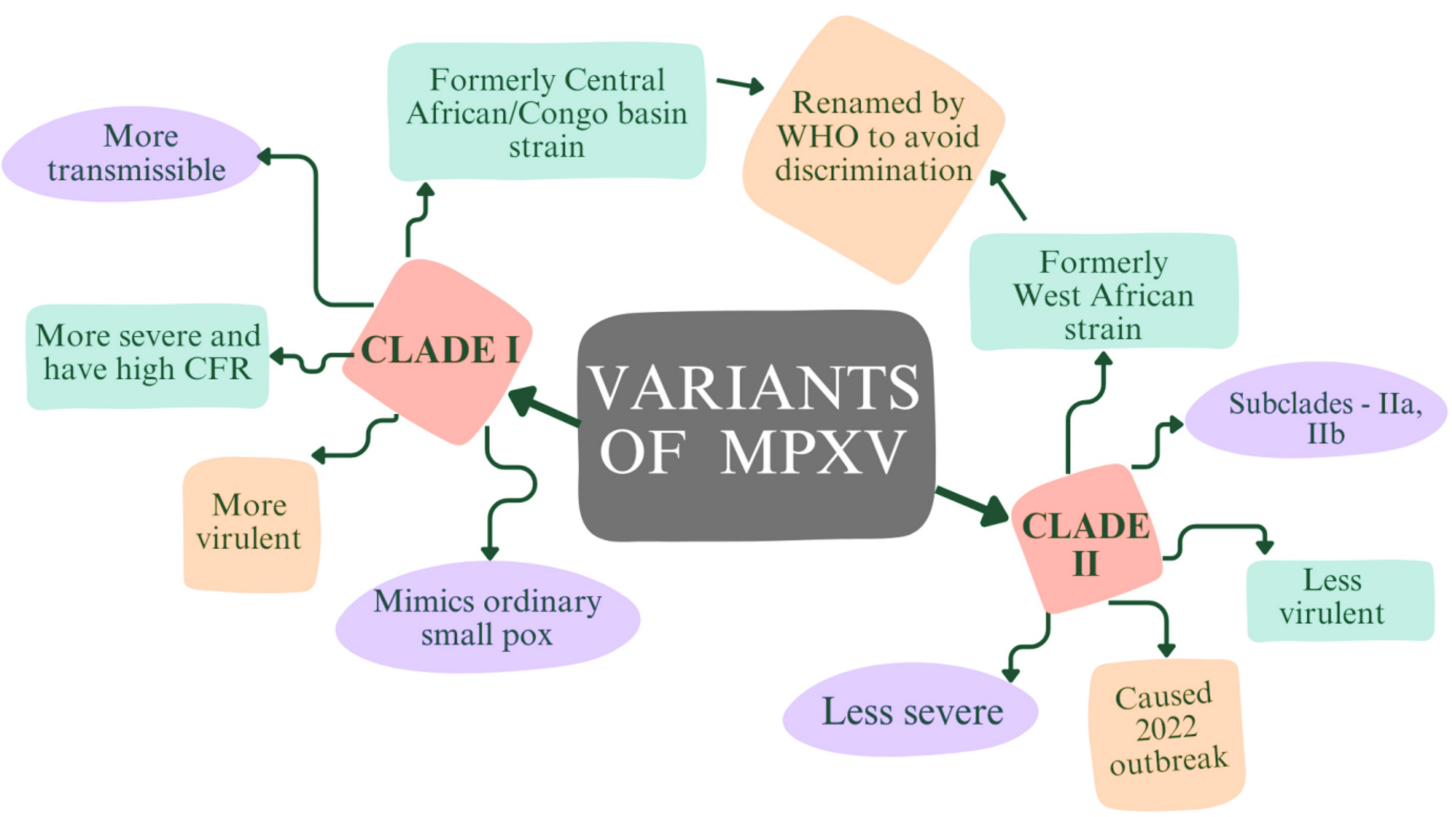
Brief description of variants of MPXV: The above concept map provides a brief description and comparison of two different genetic variants or clades of MPXV, namely Clade I and Clade II MPXV: monkeypox virus, CFR: case fatality rate, WHO: World Health Organization Created using BioRender

The MPXV is categorized into two viral clades, Clades I and II. These clades and their respective subclades, IIa and IIb, hold genetic, clinical, and geographical distinctions [34]. The MPXV clade sequences, Clade I and Clade II, differ mainly through deletions and additions in the terminal genomic portions. The Congo Basin/Central African and the West African clades were initially proposed identities for Clades I and II, respectively [34]. The WHO assembled an expert committee to suggest renaming the virus to prevent stigmatization and discrimination. Clade I was the revised name for the strain from the Congo Basin, and Clade II was for the strain from West Africa [35]. A brief description of these genetic clades was given through a concept map in Figure 3.

Clade I

Clade I was initially identified as the Central African/Congo Basin clade, representing a distinct genetic lineage. Human infections associated with Clade I resemble typical smallpox, with similar incubation and prodromal periods, characterized by symptoms like fever, headache, and malaise [34]. Clade I demonstrates higher transmissibility and virulence than Clade II. Studies emphasize that Clade I infections often lead to more severe disease presentations and a higher CFR than Clade II [34]. Clade I’s mortality rate reached up to 10%, while Clade IIa exhibited a mortality rate of less than 1% [36].

Clade II

Clade II is divided into two subclades: Clade IIa, formerly known as the West African clade, and Clade IIb, a newly recognized subgroup. Differentiating itself from Clades I and IIa, Clade IIb exhibits increased human-to-human transmission. This elevated transmission may have occurred across various global regions [34]. Clade IIb has been linked to the 2022 Mpox outbreak. Notably, severe manifestations and fatalities have occurred among immune-compromised adults with HIV in the context of Clade II MPXV infections during this outbreak. The upsurge in Clade IIb cases in 2022 is attributed to human transmission beyond Africa [36]. Disruptions have been found in many ORFs in Clade II, especially in Clade IIb, that encode genes implicated in immune evasion. These mutations may contribute to Clade II's lower virulence than Clade I [32].

Comparative genomic analysis reveals a nucleotide difference of approximately 0.55% to 0.56% between Clades I and IIa. These differences primarily occur in regions encoding significant virulence genes, likely accounting for variations in clinical severity. Clade I is estimated to contain 173 distinct functional genes, whereas Clade IIa is projected to harbor 171 unique genes. Disparities in pathogenicity between Clades I and II are attributed to differences in specific gene orthologs [35]. The two major clades, I and II, with around 0.5% genomic sequence divergence, correspond to distinct disease presentations. Retrospective investigations and experimental infections in cynomolgus monkeys have indicated that Clade I MPXV exhibits higher virulence and a higher CFR than Clades IIa/IIb [37]. Notably, MPXV variants are subject to continuous evolution.

Replication of MPXV

Since MPXV and Vaccinia virus (VACV) share over 97% of their genome sequence, the DNA replication machinery of both viruses is highly conserved, indicating that MPXV could benefit from the same research that was done on VACV [38]. Unlike most DNA viruses, which tend to replicate in the nucleus, the replication of poxviruses occurs in the cytoplasm of cells. Rather than depending on cellular proteins, they use a significant amount of their own encoded proteins during replication [39].

**Figure 4:**
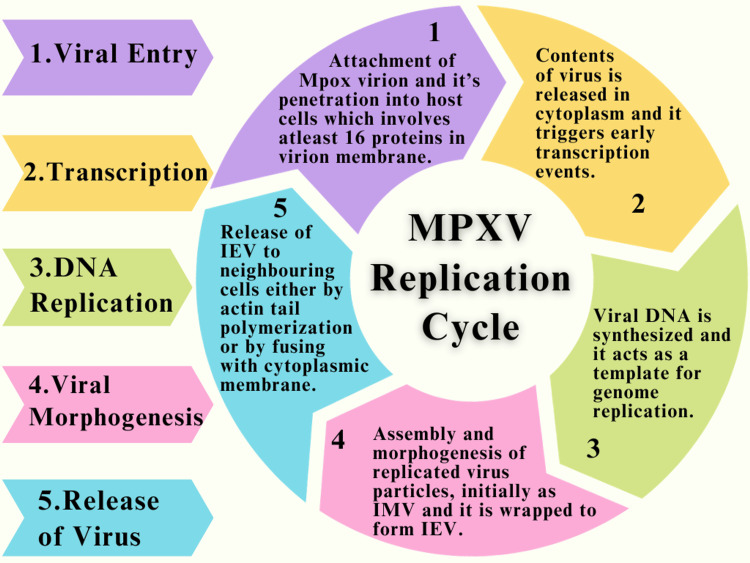
Flow diagram of replication of MPXV: The above flow chart concisely explains the process of replication of MPXV by dividing the progress into five steps, starting with viral entry into the host cells, followed by transcription events, replication of viral DNA, assembly and morphogenesis of replicated viral particles, and finally the release of IEV to the neighboring cells MPXV: monkeypox virus, DNA: deoxyribonucleic acid, IEV: intracellular enveloped viruses, Mpox: monkeypox, IMV: intracellular mature virus Created using BioRender

MPXV replicates within specialized cytoplasmic structures known as viral factories, formerly called Guarnieri bodies. These structures originate from individual infecting particles and undergo evolutionary changes during the early stages of infection. Viral factories are compact entities containing DNA encased by membranes, believed to derive from the cell's rough endoplasmic reticulum. These factories serve as focal points for essential viral processes, encompassing DNA replication, gene expression, and the production of virions [40]. The process of replication of MPXV was concisely explained through a flow chart in Figure 4.

Process of Replication

MPXV replication begins with the attachment of Mpox virion, which is most likely facilitated by cellular glycosaminoglycans and external proteins of virion on the target cell's surface [41]. Upon attachment, poxviruses penetrate the host cells through the nasopharyngeal, oropharyngeal, subcutaneous, intradermal, and intramuscular routes; at least 16 proteins in the virion membrane are involved in the entry process. Alternatively, they can directly fuse with the plasma membrane at a neutral pH or through the low-pH endosomal pathway [42].

Following entrance, the content of the densely packed core is released into the cytoplasm [43]. After that, the virus triggers early transcription events of genes, and at perinuclear locations known as viral factories, viral DNA is synthesized. The cytoplasm is where viral genes are subsequently expressed once the virus's DNA serves as a template for the genome's replication [43]. After the inoculation, MPXV replicates and spreads to various regions, which include the blood, bone marrow, lymph nodes, and other organs, due to inflammatory immune-mediated phagocytosis [3].

Mature virion (MV) and enveloped virion (EV) are the two types of particles produced by the poxvirus replication cycle. MV is composed of a single lipid bilayer, while EV has an additional external membrane. The most prevalent particles released during cell lysis are known as MVs, and they act as a mediator of transmission between hosts. Despite this, endosomes, or Golgi bodies, produce EVs. Until they are ejected, they stay connected to the host's cellular membrane and cause internal transmission inside the host [44].

MV generally reside inside the cell, but some are transferred by microtubules and end up sealed in two membranes, which are either derived from the endoplasmic reticulum or the Golgi-derived membranes. These EVs have two possible ways to leave the cell: either they fuse with the cytoplasmic membrane and become EEVs, or they can start actin polymerization, which moves the component on an actin tail toward a neighboring cell. A complex of approximately twelve non-glycosylated viral membrane proteins is necessary for the fusion of EEVs and IMVs with the cell [41].

The virus cannot propagate across long distances or from cell to cell without EVs [40]. Understanding the essential functions of genes and molecules is crucial for preventing potential pandemics caused by MPXV or other OPV family members. Investigation into these aspects could aid in the development of new strategies.

Relationship between viral replication and biological clock

The infection-biological clock is a new concept in viral infection research that emphasizes the complex link between the body's circadian cycle, immune function, and virus-host interactions. Current data suggests that circadian rhythms play an important role in viral propagation and replication, as well as influencing the host's innate and adaptive immune responses. This regulation is especially important for viral illnesses such as Mpox, which have high zoonotic and endemic potential. Understanding the impact of circadian rhythms on Mpox virus multiplication and the host's immunological response is so critical. Research has indicated that the circadian rhythm influences critical host pathways implicated in viral replication, potentially influencing the temporal and physiological aspects of Mpox virus replication. This association implies that Mpox viral load and illness severity may differ depending on the time of day [45]. However, there is still a dearth of knowledge on how these circadian systems function in Mpox infections. Further research into the relationship between circadian rhythms and Mpox viral replication should lead to more effective clinical management and treatment options, particularly during outbreaks.

Mutations of MPXV

The genetic material of poxviruses is replicated by DNA polymerases that possess the ability to proofread, leading to an estimated mutation rate of 10−5 to 10−6 mutations for each replication site [3]. Using the available MPXV sequences from 2022, Kannan et al. [46] performed a temporal analysis to find mutations. In the analysis, two variations in F8L and two in G9R were approximately 100% prevalent. While W411L first appeared in 2018 and continued to exist in 2022, the F8L mutation developed in 2022.

When compared to earlier MPXV sequences, analysis of the 2022 outbreak viral sequences in the United States showed a large number of mutations. Such mutations primarily manifest as 5′ GA-to-AA inside the apolipoprotein B mRNA editing catalytic polypeptide-like 3 (APOBEC3) patterns [3]. APOBEC3 proteins, an essential component of the vertebrate innate immune system, prevent the multiplication of external viruses by inhibiting cytosine-to-uracil deaminase activity. These proteins, which predominantly function on single-stranded DNA, have been extensively studied concerning RNA viruses, such as HIV [47].

To confirm the impact of G-to-A mutations, it is necessary to ascertain the activity of the APOBEC3 protein in various MPXV lineages. An ongoing mutational bias is evident in the fact that 5′GA-to-AA mutations accounted for most of the mutations found in expanding outbreak samples. According to Gigante et al. [47], when Clade IIb MPXV sequences were sampled between 2017 and 2022, there was a noticeable enhancement of APOBEC3-related G-to-A mutations throughout Lineage A. This indicates that the mutational effect is recurrent and dominant in the recent evolution of MPXV. The role of APOBEC3 editing in the poxvirus mutation mechanism was not understood or acknowledged prior to the 2022 outbreak.

In another study, Jones et al. [48] recently examined 47 sequences of genomes from Germany; specimens were taken from May 20 to July 4, 2022. Non-synonymous amino acid alterations were noted in comparison to the preceding MPXV outbreak. It's interesting to note that gene sequences taken from two lesions belonging to the same patient shared an initial 5′ gene duplicate. 856- nucleotides were translocated between genome termini, causing four genes close to the 3′ genome end to be completely disrupted or deleted. The virus adaptation in the unusual spread from person to person in the 2022 Mpox outbreak may have been made possible by such genome modifications in OPV, which are known to provide fitness advantages against the host’s defense mechanisms.

Recently, in 2024, an unusual and persistent Clade I Mpox epidemic in the city of Kamituga in the Eastern DRC was found and reported by Masirika et al., along with its mutational profile and whole gene annotation, through their cross-sectional cohort research. Patients of all ages with symptoms of Mpox infection who were hospitalized at Kamituga Hospital between September 2023 and the end of January 2024 were taken as subjects for the research. After isolating and sequencing the DNA from Mpox-swabbed lesions in patients, mutational profiling and phylogenetic analysis were performed. The research outcomes indicated that the Mpox epidemic in Kamituga is a new subgroup of Clade I MPXV, which they named subgroup VI. They concluded that this particular cluster of Mpox illnesses differs genetically from other Clade I outbreaks that have been documented. The necessity of continuous monitoring and the need to plan for emerging Mpox hazards in endemic areas are highlighted by these investigations [49].

Drug Resistance Due to Mutations

**Figure 5:**
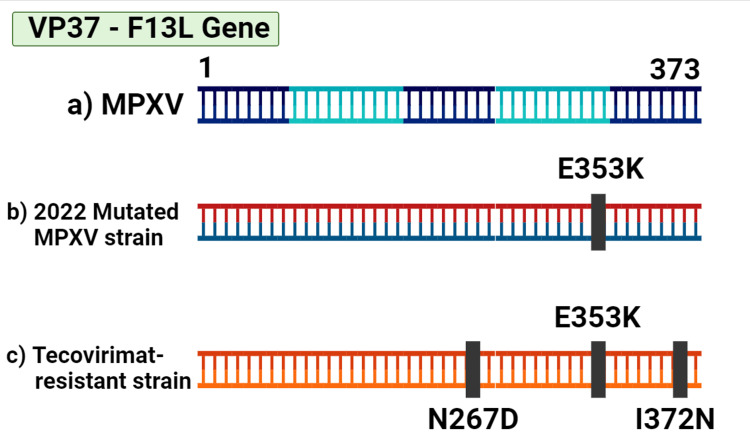
Genomic representation of mutations in MPXV: The above diagram represents the various mutations that have occurred in the F13L gene of MPXV. In the diagram, (a) indicates the early MPXV gene, (b) represents the mutated MPXV strain in 2022, and (c) demonstrates the mutation of MPXV due to tecovirimat drug resistance MPXV: monkeypox virus Created using BioRender

Amidst the global MPXV virus outbreak in 2022, tecovirimat was widely utilized for the first time in the United States. However, according to the investigation by Smith et al. [50], tecovirimat shows resistance in human Mpox cases. It is known to be caused by single amino acid mutations in the F13L gene of MPXV, which is represented in Figure 5 [51]. Thirteen new mutations and 11 known mutations that have been linked to tecovirimat resistance were found through genomic sequencing during the investigation.

Apart from tecovirimat, brincidofovir and cidofovir - two nucleoside inhibitors - are authorized for the treatment of Mpox. Changes in A314T and A684V of the VACV resulted in cidofovir resistance. The MPXV protein conserves both A314 and A684. Thus, MPXV resistance to these medications may develop via similar pathways. Since essential functional routes have already been made vulnerable to functional mutations in the viral proteins, the emergence of resistance mutations is still conceivable [46]. Among the elements that contribute to MPXV's increased transmissibility, infectiousness, and immune evasion are its mutation rate, adaptability, and genetic evolution [3].

## Conclusions

The recent global outbreak of Mpox, caused by the MPXV virus, underscores the need for ongoing research, particularly in understanding the roles of specific genes due to the virus's potential for pandemic spread. The study addresses the historical context of human Mpox in both endemic and non-endemic regions and provides up-to-date epidemiological data. It also explores the virology of MPXV, including its genetic clades, replication mechanisms, and various mutations. Emerging research highlights the significant influence of circadian rhythms on viral replication and the progression of diseases like Mpox. These findings could provide valuable insights into the ongoing control and prevention of Mpox, as well as guide future research and public health efforts. However, the study is limited by not encompassing the full genetic variability of MPXV or the complexity of host-virus interactions. Despite these limitations, this research is crucial for developing targeted strategies to combat viral threats, as understanding the interplay between circadian rhythms and viral replication could offer new avenues for intervention in managing viral diseases. Further investigation into these areas is essential to enhancing our ability to respond to future outbreaks effectively.
